# Fibrinogen–Drug Nanoparticles Achieve Durable Responses in Pancreatic and Breast Cancer Murine Models

**DOI:** 10.1021/acsptsci.6c00337

**Published:** 2026-08-21

**Authors:** Rose Razavi, Michael O’Connor, James F. Hainfeld

**Affiliations:** † 6572 Nanoprobes, Inc., Yaphank, New York 11980, United States; ‡ Penn Institute for RNA Innovation, Perelman School of Medicine, University of Pennsylvania, Philadelphia, Pennsylvania 19104, United States; § Department of Systems Pharmacology and Translational Therapeutics, Perelman School of Medicine, University of Pennsylvania, Philadelphia, Pennsylvania 19104, United States

**Keywords:** fibrinogen, nanoparticles, cancer, mice, breast cancer, pancreatic cancer

## Abstract

Inadequate targeting, short-lived therapeutic exposure, drug resistance, and toxicities limit effective cancer treatments. We developed a strategy that redirects an evolutionarily optimized clotting cascade to achieve selective, sustained drug deposition within solid tumors. A vascular disrupting agent induces tumor-specific endothelial injury, activating platelets and exposing fibrinogen-binding sites. Systemically administered fibrinogen–drug nanoparticles (FDNs) bind activated platelets with multivalent avidity, amplify thrombus formation, and become immobilized within tumor vessels, creating an intratumoral drug depot that maintains drug concentrations far above cytotoxic levels for over 10 days. A single 20 min treatment induced durable complete remissions in most mice bearing subcutaneous triple-negative breast or pancreatic tumors. This novel vascular-targeted approach enhances selective intratumoral drug localization through multivalent platelet binding, embolization, and sustained drug release to combat refractory cancers.

## Graphical abstract

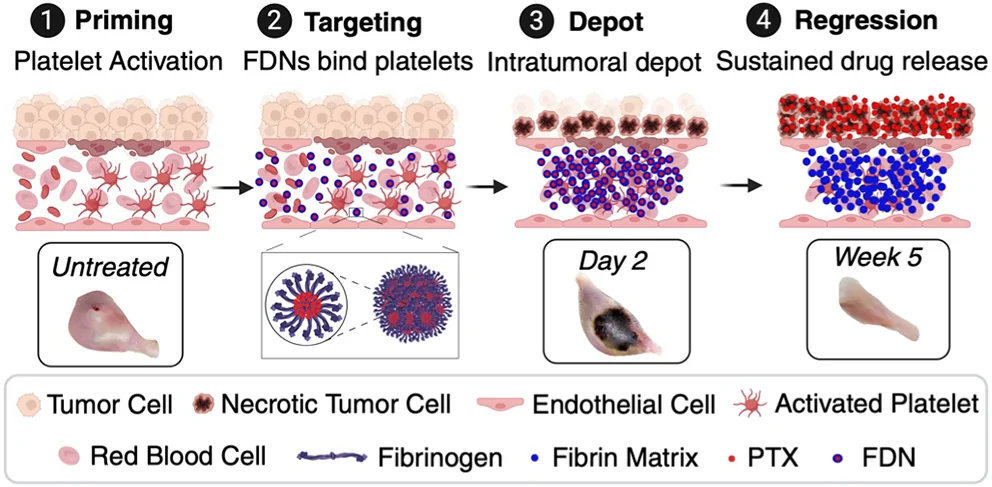

## Introduction

Despite major advances in oncology, many solid tumors remain resistant to durable control. Cytotoxic agents suffer from systemic toxicity, rapid elimination and drug resistance,^1,2^ while nanoparticle formulations—though designed to improve solubility, circulation time, and biodistribution—still deliver only a small fraction of drug to tumors. Across dozens of studies, nanoparticles achieve a median of 2.2% of the injected dose per gram of tumor,^3^ reflecting poor extravasation, limited stromal penetration, and rapid clearance.^4^ Even when nanoparticles reach tumors, ineffective release kinetics and poor cellular uptake further limit efficacy.^5^ Immunotherapies and antibody–drug conjugates provide greater specificity,^5^ yet many tumors remain immunologically cold,^6,7^ rapidly develop resistance, or lack the required surface markers.^8^ Collectively, these limitations underscore the persistent challenge of achieving high, sustained therapeutic exposure within solid tumors.

Several groups have previously explored strategies that leverage coagulation biology or therapy-induced vascular changes to enhance nanoparticle accumulation in tumors. Prior approaches have used coagulation activation or vascular disruption to enhance nanoparticle accumulation.^9,10^ These studies demonstrated that therapeutic modulation of the tumor microenvironment can enhance nanoparticle localization. However, these prior systems primarily relied on ligand-mediated targeting or externally induced signaling mechanisms and did not directly harness fibrinogen—the endogenous substrate of platelet aggregation—to amplify thrombosis and create a sustained intratumoral drug reservoir. In contrast, the strategy described here uses fibrinogen itself as both the nanoparticle scaffold and the targeting element, enabling direct multivalent binding to activated platelet GPIIb/IIIa receptors and incorporation into the growing platelet–fibrin network. This design allows the drug-loaded particles to become physically integrated into tumor thrombi, producing both vascular embolization and prolonged intratumoral drug release.

To overcome anatomical and physiological barriers, we turned to an evolutionarily optimized biological system already specialized for rapid, localized polymerization: the clotting cascade.^11^ When a blood vessel is injured, platelets rapidly activate, expose the high-affinity integrin GPIIb/IIIa,^12^ bind fibrinogen (Figure 1B), and assemble a multivalent platelet-fibrin matrix that seals vascular breaches with remarkable speed and strength. This process relies on high-avidity binding, explosive amplification, and site-specific polymer formation, precisely the attributes required for effective tumor-localized drug delivery.

**Figure 1:**
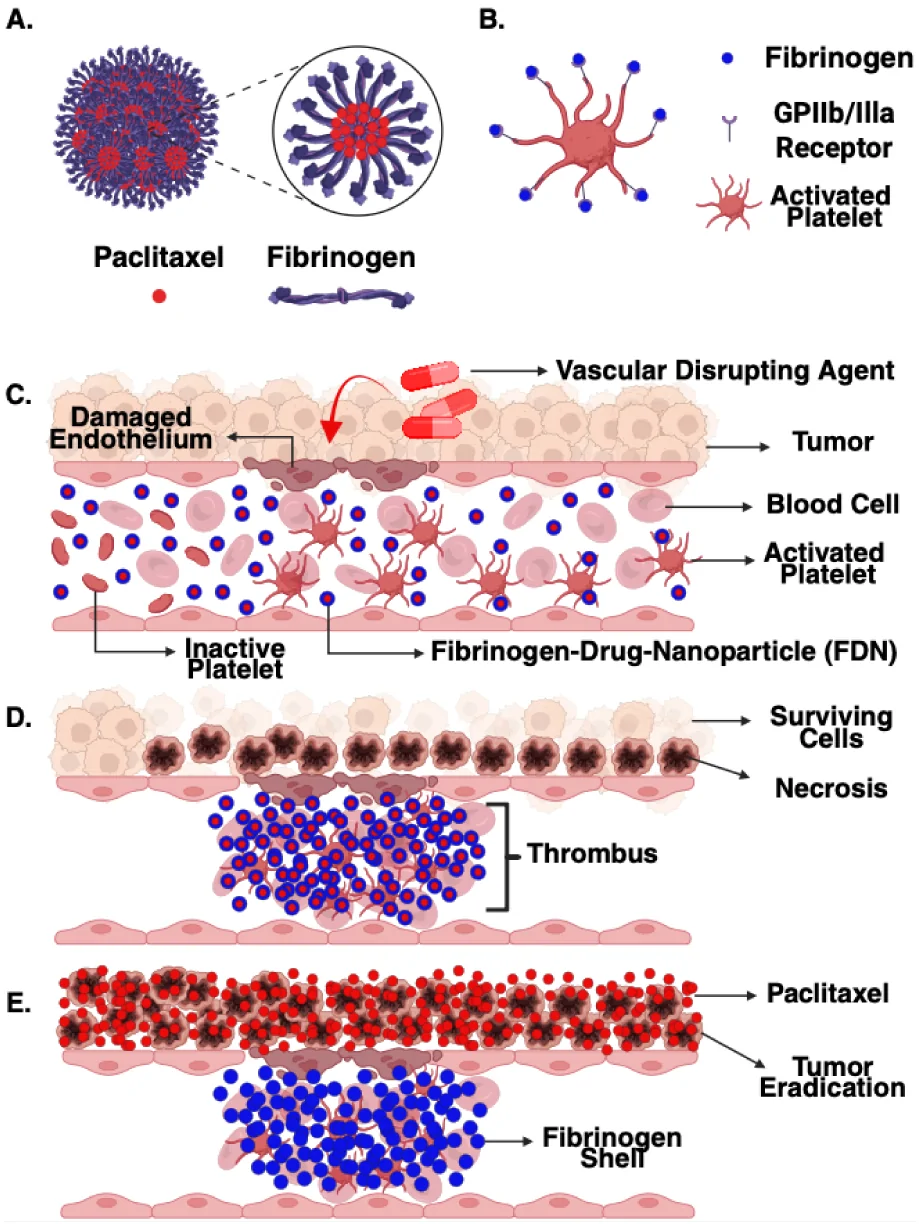
Mechanism of fibrinogen–drug nanoparticle (FDN) therapy (A) schematic structure of an FDN, composed of a drug (e.g., paclitaxel) encapsulated within a fibrinogen-based nanoparticle. (B) Activated platelets expose the integrin GPIIb/IIIa, which binds the fibrinogen component of FDNs with high avidity. (C) A vascular disrupting agent selectively injures tumor endothelium, triggering local platelet activation and enabling intravascular capture of circulating FDNs. (D) Multivalent FDN binding amplifies platelet aggregation and fibrin polymerization, forming an obstructive thrombus that embolizes tumor vessels and induces downstream necrosis. (E) Immobilized FDNs act as a drug-releasing depot, delivering cytotoxic drugs over extended periods to eradicate surviving tumor cells. Created in BioRender. Razavi, R. (2026) https://BioRender.com/j28klob.

We hypothesized that if a tumor could be primed to transiently mimic vascular injury, activated platelets could be exploited as a drug-capturing surface. This approach integrates three coordinated steps (Figure 1C–E): (1) targeted endothelial damage to activate platelets, (2) fibrinogen–drug nanoparticle (FDN) binding that amplifies thrombosis and embolizes tumor vessels, and (3) sustained intratumoral drug release from the resulting platelet–fibrin scaffold. Together, these steps convert the tumor’s own vasculature into a localized delivery and destruction platform.

Mechanistically, FDNs out-compete endogenous fibrinogen for GPIIb/IIIa binding; their multivalency and larger size drive more extensive platelet cross-linking and more robust vessel occlusion, inducing downstream necrosis and enabling prolonged intratumoral drug exposure. This approach achieves sustained intratumoral drug exposure and durable tumor eradication.

## Results

### Construction and Characterization of Fibrinogen–Drug Nanoparticles

Sonicating fibrinogen with paclitaxel produced uniform 183 ± 8.9 nm nanoparticles with low polydispersity (PDI, 0.18 ± 0.04, Figure 2A) and high encapsulation efficiency (91.7 ± 4.0%), corresponding to 0.076 mg PTX per mg fibrinogen (7.6 wt %). Sonication is known to induce partial protein unfolding, exposing internal hydrophobic residues and increasing surface hydrophobicity. These conformational changes are thought to promote stable noncovalent association of hydrophobic paclitaxel with fibrinogen while simultaneously driving protein aggregation, thereby providing a plausible mechanism for fibrinogen–drug nanoparticle formation.^13^ Transmission electron micrographs of the fibrinogen–drug nanoparticles (FDNs) corroborated the particle sizes measured by dynamic light scattering (Supplementary Figure S2). Given their larger size and multimeric structure (Figure 1A), FDNs were expected to undergo more extensive fibrin polymerization than free fibrinogen once exposed to thrombin. To evaluate this, we used the Clauss assay, in which thrombin converts fibrinogen to fibrin^14^ and the resulting increase in turbidity reflects polymer formation over time.^15^ Consistent with this prediction, FDNs generated ∼30-fold greater fibrin polymerization than an equivalent amount of free fibrinogen (Figure 2B), demonstrating markedly enhanced incorporation into clot formation and supporting their ability to amplify thrombosis in vivo.

**Figure 2:**
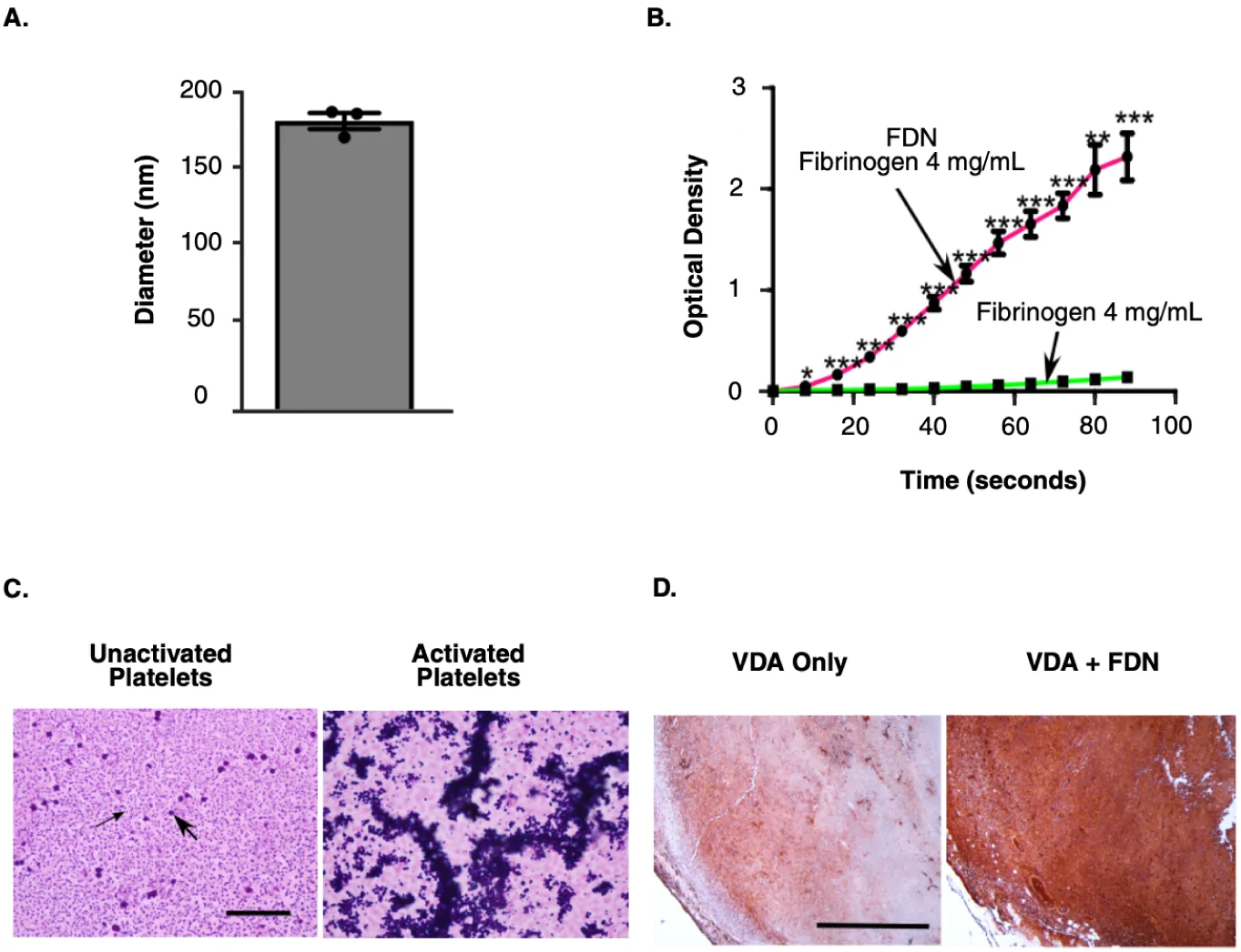
Fibrinogen–drug nanoparticles retain coagulation activity, promote platelet aggregation, and enhance intratumoral fibrinogen deposition following vascular disruption. (A) Hydrodynamic diameter of fibrinogen–drug nanoparticles (FDNs), demonstrating uniform nanoparticle size. Bars represent mean ± SEM. (B) Clauss fibrin polymerization assay comparing free fibrinogen and FDNs containing an equivalent fibrinogen concentration (4 mg/mL) following thrombin activation. FDNs exhibit markedly enhanced polymerization kinetics and optical density relative to free fibrinogen, indicating preserved and amplified coagulation activity (*p* < 0.0001). (C) Platelet aggregation in platelet-rich plasma following exposure to FDNs. Left, platelets isolated in the presence of prostaglandin E1 (aggregation inhibited) show no aggregation upon FDN addition. Right, ADP-activated platelets rapidly form large aggregates upon FDN exposure. Large arrow indicates red blood cell; small arrow indicates platelet. Scale bar, 20 μm. (D) Immunohistochemical staining for fibrinogen (brown) in EMT6 tumors 24 h after treatment with vascular disrupting agent (VDA) alone or VDA combined with FDNs. VDA + FDN treatment results in extensive fibrinogen accumulation throughout tumor tissue compared with VDA alone, indicating enhanced nanoparticle deposition at sites of vascular damage. Scale bar, 0.5 mm. Created in BioRender. Razavi, R. (2026) https://biorender.com/94cuaim.

### FDNs Bind Selectively to Activated Platelets

FDNs were next evaluated for their ability to bind specifically to activated platelets. To do this, platelet-rich plasma (PRP) was isolated from mouse blood and used in two parallel assays. In one condition, prostaglandin E1 was added to PRP to inhibit platelet activation; when mixed with FDNs, no binding or aggregation was observed (Figure 2C), indicating minimal interaction with quiescent platelets. In contrast, when PRP was activated with ADP before the addition of FDNs, extensive and immediate platelet aggregation occurred (Figure 2C). These findings demonstrate that FDNs selectively interact with activated platelets—consistent with GPIIb/IIIa-mediated recognition—and support their ability to target platelet activation sites generated after tumor endothelial injury.

### VDA-Induced Platelet Activation Enables Intratumoral Capture of Fibrinogen Constructs

Administration of the vascular disrupting agent DMXAA selectively damaged the tumor vasculature, leading to rapid platelet activation. Immunohistochemistry revealed substantially greater fibrinogen deposition throughout tumors treated with VDA + FDNs than with VDA alone, demonstrating efficient intratumoral capture of the nanoparticles. When tumors were treated with VDA alone, fibrinogen deposits were typically restricted to the tumor periphery, where VDAs preferentially disrupt angiogenic endothelium.^16–18^ Surprisingly, when FDNs were used in combination with VDA, heavy fibrinogen deposits were observed not only at the periphery but also deeper within the tumor mass (Figure 2D). This widespread intratumoral deposition is consistent with multivalent FDN–platelet interactions amplifying thrombus propagation, allowing clot formation to extend into deeper tumor regions.

### Fibrinogen–Paclitaxel Nanoparticles (FDN-PTX) Drive Durable Responses in a Triple-Negative Breast Cancer Model

Within 24–48 h of VDA + FDN treatment, tumors became visibly black and necrotic, far exceeding the mild peripheral necrosis observed with VDA alone (Figure 3A). This therapy was accompanied by transient weight loss over the first 5 days, after which animals rapidly recovered to baseline (Figure 3G). These highly necrotic tumors regressed over subsequent weeks by 4–5 weeks after treatment, leaving no detectable residual tumor (Figure 3B, C). This vascular shutdown produced robust therapeutic efficacy across a wide range of tumor sizes (145–1020 mm^3^; mean 543 mm^3^, Figure 3F). A single treatment resulted in complete tumor regression in 14/16 mice with no recurrence over 200 days (Figure 3D, E).

**Figure 3:**
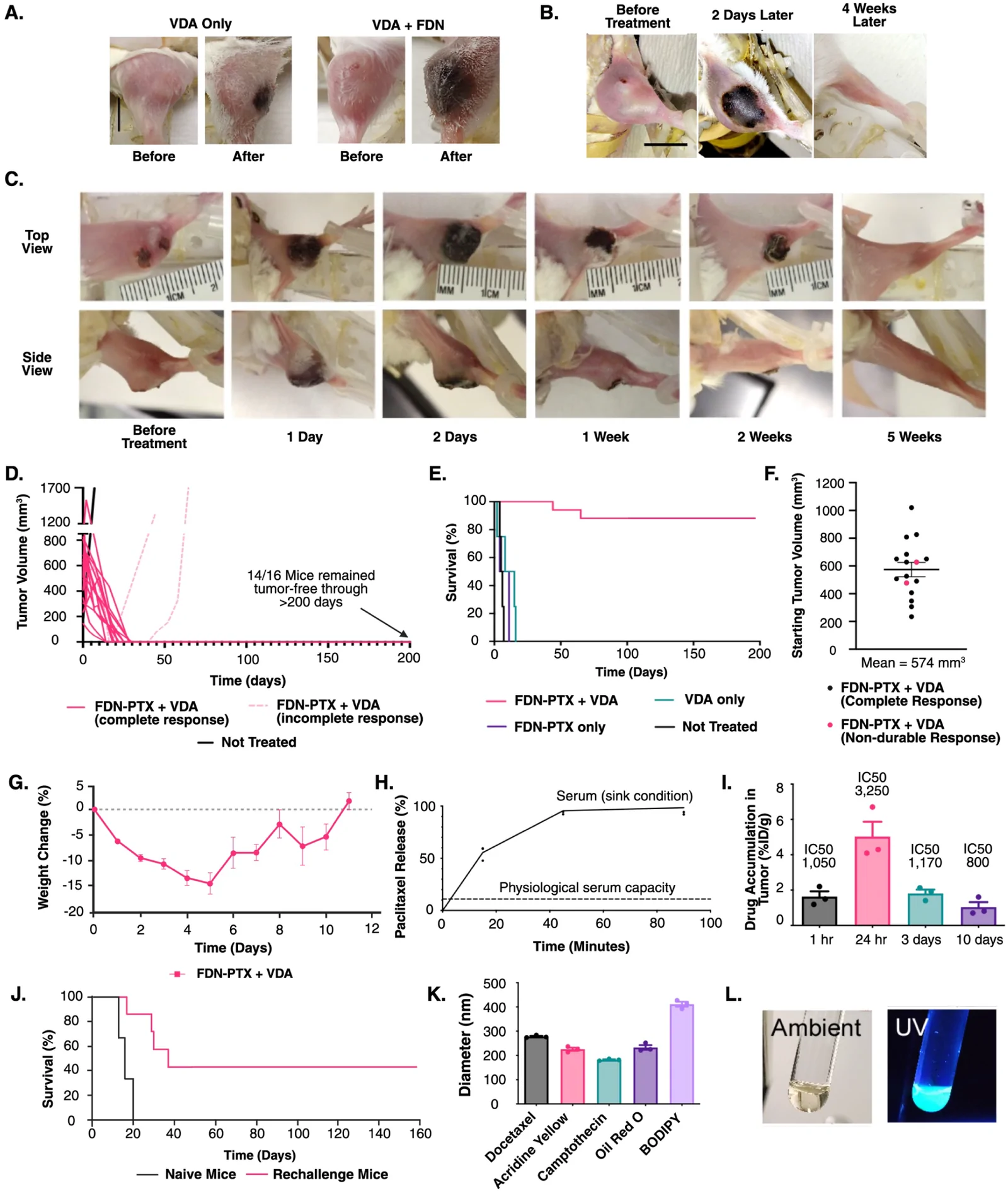
Fibrinogen–drug nanoparticles (FDNs) combined with vascular disrupting agents induce rapid tumor necrosis, durable cures, and long-term antitumor immunity in EMT6 TNBC. (A) Representative images of EMT6 triple-negative breast tumors before and 24 h after treatment with vascular disrupting agent (VDA) alone or VDA combined with fibrinogen–drug nanoparticles (FDNs). Combined VDA + FDN treatment induces markedly greater tumor necrosis compared with VDA alone. Scale bar, 0.5 cm. (B) Longitudinal photographs of a large 1020 mm^3^ EMT6 tumor before treatment, 2 days after a single VDA + FDN treatment, and 4 weeks later, showing complete tumor elimination without regrowth. Scale bar, 1 cm. (C) Serial top- and side-view images illustrating treatment response over time following VDA + FDN therapy. Extensive tumor necrosis and scab formation occur within days, followed by progressive tumor shrinkage and complete resolution by 5 weeks, with restoration of normal tissue appearance and no detectable recurrence. (D) Individual tumor volume trajectories following VDA + FDN therapy. Each line represents one mouse. Fourteen of 16 mice exhibited complete and durable tumor regression, remaining tumor-free for >200 days. Typical tumor growth of an untreated control is shown for comparison. (E) Kaplan–Meier survival analysis of EMT6 tumor–bearing mice. VDA + FDN treatment resulted in 88% long-term survival (>200 days), whereas all control groups (untreated, FDN only, or VDA only) succumbed within 20 days. Survival of the VDA + FDN group was significantly improved compared with all controls (log-rank Mantel–Cox test, *p* < 0.001). Group sizes: VDA + FDN, *n* = 16; untreated, *n* = 4; VDA only, *n* = 4; FDN only, *n* = 4. (F) Distribution of starting EMT6 tumor volumes at the time of treatment, demonstrating effective tumor eradication across a broad range of initial tumor sizes. (G) Body weight changes following VDA + FDN therapy, showing transient weight loss with recovery to baseline within ∼11 days, indicating tolerable systemic toxicity. (H) Paclitaxel (PTX) release from FDNs into serum under sink conditions. PTX is released nearly to completion within 1 h. The dashed line indicates the estimated low release into the blood pool after injection due to poor serum capacity to solubilize a hydrophobic drug. (I) Tumor accumulation of PTX following VDA + FDN administration, measured at 1 h, 24 h, 3 days, and 10 days postinjection, demonstrating rapid tumor delivery and sustained intratumoral drug levels. (J) Rechallenge experiment demonstrating treatment-induced antitumor immunity. Mice cured by VDA + FDN therapy either rejected secondary tumor implantation (43%) or exhibited significantly delayed tumor growth compared with naïve controls (log-rank Mantel–Cox test, *p* = 0.0048). (K) Hydrodynamic diameters of fibrinogen nanoparticles loaded with diverse small-molecule cargos, demonstrating modular nanoparticle construction. (L) Representative images of fluorescently labeled fibrinogen nanoparticles under ambient light and UV illumination. Created in BioRender. Razavi, R. (2026) https://biorender.com/94cuaim.

### Sustained High Intratumoral Paclitaxel Levels Following FDN Delivery

FDNs delivered ∼5%ID/g paclitaxel at 24 h, exceeding the IC50 by ∼3,000-fold and remaining above cytotoxic levels for >10 days, yielding an AUC of 5,620 h·μg/g (Figure 3I). When initially injected, only ∼11% of the paclitaxel can be released into the serum due to its poor solubility in serum^19^ (Figure 3H). Under sink conditions, paclitaxel release from fibrin-clotted FDNs was rapid reaching completion within ∼1 h (Figure 3H). This concentration-ratio dependent release, combined with the physical immobilization of FDNs within tumor thrombi, provides a mechanistic explanation for the sustained high drug levels in tumors.

### FDN Therapy Induces Tumor-Specific Immunity

Immunotherapy is a highly promising strategy for long-term tumor control; however, many tumors exhibit an immunologically “cold” phenotype and fail to respond.^6,7^ To determine whether FDN therapy stimulates a protective antitumor immune response, mice that had been treated and cured were rechallenged with tumor cells in the contralateral leg, and tumor growth was monitored (Figure 3J). Notably, 43% (3 of 7) of previously treated mice showed complete absence of tumor growth, while an additional 4 of 7 exhibited substantially slower tumor progression compared to naïve controls, indicating that FDN therapy induces an adaptive immune response capable of suppressing tumor recurrence.

### Platform Versatility for Multiple Hydrophobic Agents

Since paclitaxel readily incorporates into fibrinogen, we next evaluated five additional hydrophobic compounds to assess platform versatility. Multiple hydrophobic compounds, including chemotherapeutics, dyes, and fluorophores, formed fibrinogen-based nanoparticles comparable to FDN-PTX (Figure 3K), demonstrating platform versatility. This versatility suggests broad translational potential, enabling adaptation of the platform for both therapeutic and diagnostic applications across multiple disease contexts.

### Fibrinogen–Docetaxel Nanoparticles (FDN-DTX) Drive Durable Responses in a Desmoplastic Pancreatic Cancer Model

Having established robust efficacy in the EMT6 breast cancer model and demonstrated compatibility with multiple hydrophobic payloads, we next evaluated the platform in a KPC pancreatic cancer model, a clinically relevant and therapeutically refractory tumor context characterized by dense desmoplasia and dysregulated coagulation biology. The KPC model closely recapitulates key genetic drivers and pathological features of human pancreatic ductal adenocarcinoma (PDAC), including activating Kras and mutant p53 signaling.^20–22^

Initial evaluation of paclitaxel-loaded fibrinogen drug nanoparticles (FDN-PTX) in KPC tumors demonstrated measurable antitumor activity but produced heterogeneous outcomes. Tumor growth curves revealed substantial variability in therapeutic response, with 2 of 13 treated animals achieving complete tumor regression while others exhibited partial or transient responses followed by tumor progression (Supplementary Figure 1A). Consistent with these observations, Kaplan–Meier survival analysis showed improved survival relative to untreated controls but incomplete long-term tumor control (Supplementary Figure 1B).

To determine whether an alternative taxane payload could improve therapeutic performance in this tumor context, we evaluated docetaxel (DTX), a related taxane with higher affinity for tubulin,^23^ using a modified formulation procedure that yielded a larger fibrinogen–drug nanoparticle with reduced colloidal stability incorporating DTX (FDN-DTX) (433 ± 137 nm with a PDI of 0.21 ± 0.06) immediately after preparation) that increased in size to 575 ± 124 nm with a PDI of 0.29 ± 0.05 after 20 min.

With this optimized preparation, VDA-primed delivery of FDN-DTX produced rapid tumor regression and durable complete responses in almost all treated animals (8/9). Tumors became undetectable by caliper measurement within approximately 12–25 days following treatment and remained absent throughout the duration of the study. Mean tumor growth curves (±SEM) demonstrate complete and sustained tumor clearance in the FDN-DTX + VDA group, whereas untreated and VDA-only control groups exhibited progressive tumor growth with overlapping trajectories (Figure 4A). Kaplan–Meier analysis further demonstrated durable survival benefits, with 8/9 treated animals remaining tumor-free beyond 200 days of observation (Figure 4B). Treatment caused transient ∼10–12% weight loss followed by recovery (Figure 4C). Therapeutic responses occurred across a broad range of tumor volumes (100–>1000 mm^3^; mean 454 mm^3^; Figure 4D). Together, these findings demonstrate that docetaxel-based fibrinogen nanoparticles markedly enhance therapeutic efficacy in KPC pancreatic tumors.

**Figure 4:**
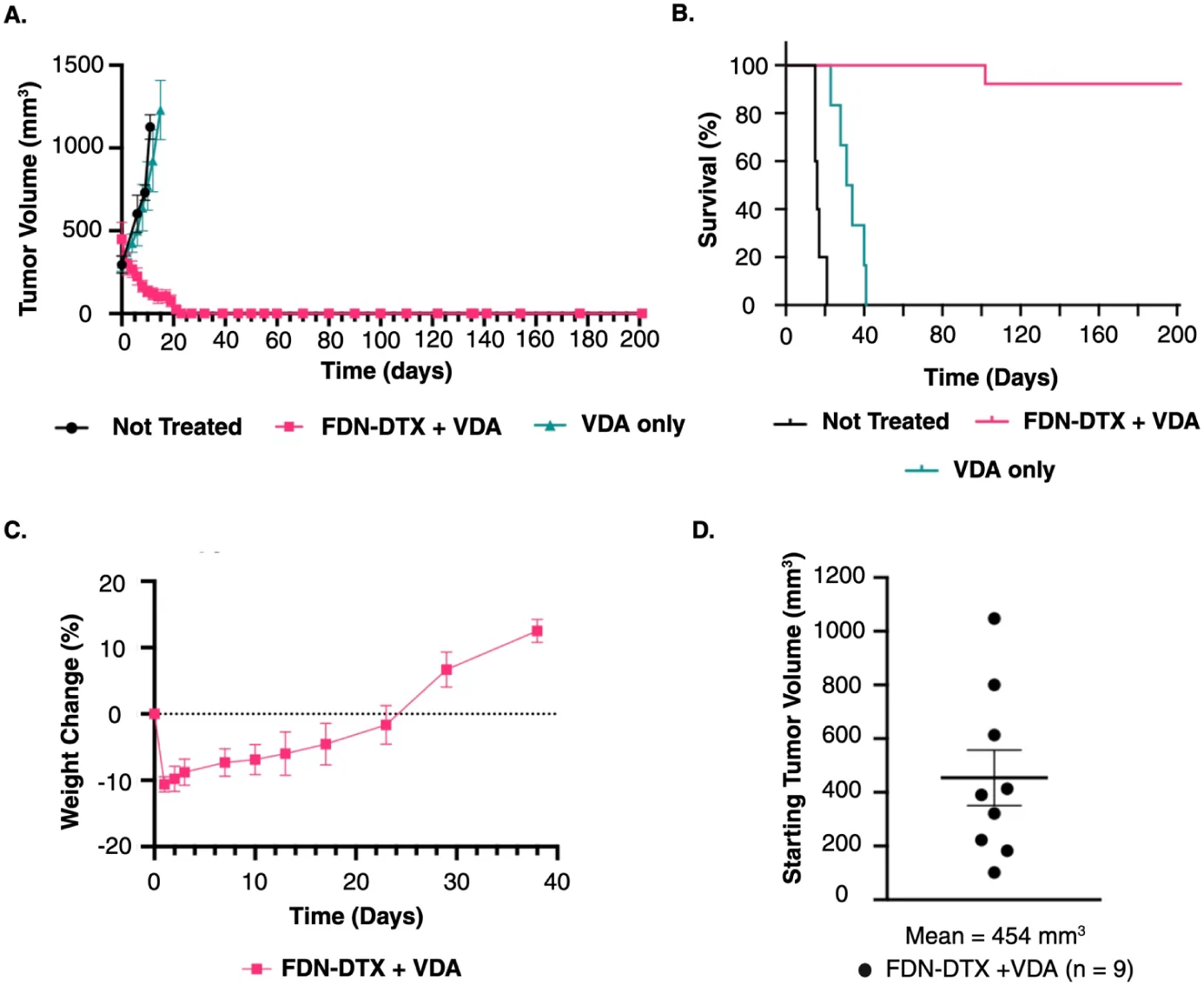
Fibrinogen–docetaxel nanoparticles eradicate KPC pancreatic tumors following vascular priming. (A) Tumor volume over time in KPC pancreatic tumor–bearing mice treated with the indicated conditions: untreated (*n* = 5), vascular disrupting agent (VDA) alone (*n* = 6), or VDA + FDN-DTX (*n* = 9). Combination therapy produced rapid tumor regression and complete disappearance of tumors within ∼12–25 days, whereas untreated and VDA-only groups showed progressive tumor growth. Data represent mean ± SEM. (B) Kaplan–Meier survival analysis of treated mice. VDA + FDN-DTX produced durable survival, with 8/9 treated animals remaining tumor-free throughout the observation period (>200 days), whereas untreated controls succumbed rapidly to tumor progression. (C) Body weight changes following treatment. VDA + FDN-DTX caused a transient decrease in body weight (∼10–12%) followed by recovery to baseline levels. (D) Distribution of starting tumor volumes at treatment initiation in the VDA + FDN-DTX cohort (*n* = 9). Tumors ranged from ∼100 to >1000 mm^3^ (mean = 454 mm^3^), demonstrating responses across a wide range of initial tumor sizes. Created in BioRender. Razavi, R. (2026) https://BioRender.com/mgjdehh.

## Discussion

This study presents a fundamentally different strategy for cancer drug delivery: hijacking an evolutionarily optimized hemostatic cascade to concentrate cytotoxic agents directly within tumor vasculature. Unlike conventional nanoparticle systems (typically optimized to be 50–200 nm to facilitate extravasation and tumor penetration^24,25^ ), FDNs exploit the exposed activated platelets within tumor blood vessels, eliminating the need for interstitial transport and allowing the use of larger, aggregation-capable constructs. Larger particle–platelet complexes increase embolic potential, enhancing vessel occlusion.

Although protein–drug nanoparticles have previously been described, the primary innovation of the present study lies not in the nanoparticle composition itself but in the biological targeting strategy. By exploiting transient vascular injury and platelet activation, fibrinogen–drug nanoparticles become selectively localized and retained within tumor vasculature, where they form a sustained intratumoral drug depot.

Transmission electron micrographs of the FDNs were consistent with the particle sizes measured by dynamic light scattering, but nanoparticle substructure was difficult to delineate because the density of docetaxel or paclitaxel (1.37 g/cm^3^) is very similar to that of protein (1.35 g/cm^3^). However, studies of albumin–paclitaxel nanoparticles prepared using a similar sonication method have reported a drug-core/protein-shell organization, suggesting that a comparable structure may also exist in FDNs, although this remains to be confirmed experimentally. Despite this limitation, the primary objective of the present work was to demonstrate that platelet-mediated biological targeting can produce prolonged intratumoral drug exposure and durable therapeutic responses.

The formulation process also minimizes potential contributions from the polyethylene glycol (PEG 200) vehicle used during nanoparticle preparation. PEG 200 was selected because of its excellent ability to solubilize hydrophobic taxanes and its established biocompatibility. During sonication, paclitaxel becomes noncovalently associated with partially unfolded fibrinogen, whereas the PEG vehicle is expected to remain largely in the aqueous phase rather than being incorporated into the nanoparticle. Consequently, only minimal residual PEG is anticipated in the final formulation. Moreover, the maximum administered PEG dose (approximately 1.8 mL/kg) is substantially below the reported intravenous LD50 in rodents (>10 mL/kg), suggesting that any residual PEG would be unlikely to contribute meaningfully to systemic toxicity under the conditions used in this study.^26^

Fibrinogen–platelet interactions drive this mechanism with exceptional affinity. The fibrinogen–GPIIb/IIIa interaction (*K*_d_ ≈ 15 nM), comparable to high-affinity antibodies,^27,28^ becomes effectively irreversible through multivalent binding.^27,29^ This interaction initiates platelet activation and aggregation,^30^ amplifying thrombus formation. Resulting ischemia leads to apoptosis and necrosis,^31^ providing an advantage over conventional therapies that rely primarily on apoptosis;^32^ necrosis is particularly effective against tumor cells that have evolved apoptosis resistance.

This embolic trapping creates a stable, long-acting drug reservoir. FDNs delivered 5%ID/g paclitaxel at 24 h, substantially higher than many reported delivery systems, including Abraxane (∼0.1%ID/g^100^ and liposomes (∼0.06%ID/g).^54^ Although modern antibody–drug conjugates can reach ∼45%ID/g,^5^ the critical distinction lies in sustained local exposure. With FDNs, paclitaxel levels remained above the IC50 for >10 days, resulting in an AUC of 5,620 h·μg/g, nearly 100-fold greater than values typically reported for antibody-drug conjugates.^33^ Importantly, prolonging paclitaxel exposure from 24 to 72 h has been shown to increase cytotoxicity by up to 200-fold.^34^ Such prolonged retention is facilitated by nanoparticle entrapment within platelet–fibrin matrices, limiting systemic clearance and enabling slow release precisely within the tumor microenvironment. Paclitaxel’s limited serum solubility restricts systemic release, while intratumoral retention (>10 days; Figure 3I) and fibrin-mediated entrapment enable sustained local drug exposure.

The durable eradication of aggressive KPC pancreatic tumors further highlights the strength and generalizability of this approach. Even large established tumors exceeding 1000 mm^3^ were eliminated following VDA-primed FDN–DTX treatment, demonstrating that this strategy can overcome the highly desmoplastic and therapy-resistant microenvironment characteristic of pancreatic ductal adenocarcinoma. The rapid and complete responses observed across treated animals suggest that coupling vascular disruption with fibrinogen-mediated drug deposition enables highly efficient intratumoral drug retention and associated cytotoxicity, even in tumor types traditionally resistant to chemotherapy. These findings support the potential of this platform, particularly in combination with vascular priming strategies, for application across multiple solid tumor types.

This vascular-targeted approach offers multiple advantages. Because it does not rely on tumor-specific biomarkers, it has broad applicability across cancer types. In addition, targeting endothelial cells helps circumvent multidrug resistance, as these cells are less prone to acquiring mutations than tumor cells.^35^ Chemotherapy fails in a large fraction of cases, often due to the development of tumor drug resistance.^36^ The brief single treatment in our approach may avoid drug resistance that typically evolves after extended multiple treatments.

Although DMXAA reached phase III clinical testing, it ultimately failed to improve overall survival,^37^ likely due to two key limitations. First, DMXAA functions as a Stimulator of Interferon Genes (STING) agonist in mice but not in humans.^37^ Second, although DMXAA reduced tumor blood flow by up to 66%,^38^ residual tumor cells at the periphery survived by obtaining oxygen and nutrients from adjacent normal vasculature, an effect known as the “viable rim” phenomenon.^39^ In contrast, the FDN strategy may overcome these limitations by coupling vascular disruption with prolonged intratumoral drug release, ensuring cytotoxic exposure extends to peripheral tumor regions. Thus, while DMXAA monotherapy is not clinically viable, the vascular disrupting property may still be leveraged using alternative vascular-disrupting agents or synthetic STING agonists in combination with the FDN platform.^40,41^ Vascular disruption can also be achieved by radiation,^42^ heat,^43^ mechanical stress^44^ (e.g., lithotripsy), or other means. Importantly, the therapeutic concept described here is not dependent on DMXAA itself, but rather on transient vascular disruption and platelet activation to establish a tumor-selective drug depot. Consequently, clinically investigated vascular disrupting agents or human-active STING agonists could potentially be substituted without altering the underlying targeting mechanism.^45^

Importantly, EMT6 tumors are considered immunologically “cold” and poorly responsive to immune activation.^46^ However, our rechallenge data indicate that FDN therapy may induce immunological memory: 43% (3/7) of cured mice exhibited complete tumor rejection upon contralateral rechallenge, and 4/7 showed markedly slower tumor growth, suggesting the generation of a sustained adaptive response. Necrotic tumor cell death likely contributes via release of inflammatory cytokines and immunostimulatory factors,^32^ potentially enhancing antitumor immunity and facilitating abscopal-like effects.^47,48^

Personalization requirements often limit access to advanced cancer therapies; however, about 90% of all cancers rely on neovascularization,^49^ making this strategy broadly relevant. Additional hydrophobic agents—including camptothecin, docetaxel, Oil Red O, acridine yellow and BODIPY—successfully formed nanoparticles like FDN–paclitaxel (Figure 3K), demonstrating formulation extensibility.

While highly promising, this approach carries potential safety concerns that warrant further investigation. Because the clotting system is co-opted for drug targeting, there is a risk of off-target thrombotic events, which could lead to adverse vascular outcomes such as stroke or myocardial infarction. Fibrin deposition is also implicated in several pathological conditions,^50^ and patients experiencing extensive tissue damage, such as burn victims, may exhibit hypercoagulability,^51^ highlighting the importance of careful toxicity evaluation. Animal clinical signs and body weight are commonly used as early indicators of systemic toxicity.^52^ In the present study, animals exhibited only transient weight loss (>5%) followed by recovery to baseline body weight and normal behavior, suggesting tolerability under the conditions tested. Nevertheless, comprehensive toxicological evaluation, including hematology, serum clinical chemistry, coagulation testing, and histopathological analysis, will be important to further characterize the safety profile of this therapeutic approach.

The platform may be extended using alternative hydrophobic drugs or platelet-targeting ligands and integrated with existing nanoparticle systems or vascular-priming strategies.

A surprising result was that only a single, simple, noninvasive ∼20 min treatment was necessary to fully eradicate many tumors compared to the extensive, multiple, prolonged and often less effective treatments currently available. We attribute this to the multifunctionality of fibrinogen–drug nanoparticles, which combine avid targeting, polymerization and stabilization within tumors, vessel embolization, sustained drug release, and biodegradability.

Together, these results establish a clinically translatable drug delivery platform that converts tumor vasculature into a long-acting intratumoral drug depot. By co-opting evolution’s fastest polymerizing system, this strategy integrates vascular embolization, sustained chemotherapy delivery, and immune activation into a unified therapeutic approach for drug-resistant solid tumors.

## Conclusion

We present a therapeutic strategy that harnesses part of the clotting cascade to eradicate tumors. By combining tumor-selective vascular disruption with fibrinogen–drug nanoparticles, this strategy triggers a self-amplifying intratumoral thrombosis that both blocks tumor blood flow and locks in a long-acting drug reservoir. These coordinated steps—(1) targeted endothelial injury, (2) nanoparticle-driven platelet capture and vessel embolization, and (3) sustained intratumoral cytotoxic drug release—convert the tumor vasculature into a therapeutic delivery and destruction platform.

Using this approach, fibrinogen–taxane nanoparticles produced durable eradication of aggressive solid tumors in two preclinical models. In triple-negative breast cancer (EMT6), a single, noninvasive treatment combining DMXAA with fibrinogen–paclitaxel nanoparticles induced extensive tumor necrosis and sustained drug exposure for more than 10 days, resulting in complete tumor elimination in most animals. Extending the platform to a highly desmoplastic KPC pancreatic cancer model using fibrinogen–docetaxel nanoparticles produced rapid and durable tumor clearance even in large established tumors.

Together, these findings establish a first-in-class vascular-targeted drug delivery strategy that converts tumor vasculature into a long-acting intratumoral drug depot, offering a broadly applicable platform for improving therapeutic localization, prolonging intratumoral drug exposure, and enhancing treatment efficacy across solid tumors.

## Materials and Methods

### Materials

Fibrinogen (Fib 1, plasminogen-depleted; bovine and human) was obtained from Enzyme Research Laboratories (South Bend, IN). Docetaxel (DTX) and paclitaxel (PTX) were purchased from LC Laboratories (Woburn, MA). DMXAA was obtained from Tokyo Chemical Industry Co. (D5235) or InvivoGen (tlrl-dmx) and prepared as a 2 mg/mL solution in 0.1 M Tris buffer (pH 8.0). Polyethylene glycol (PEG 200, MW) was purchased from Thermo Fisher Scientific (B21918). Poly-l-lysine (PB920), ADP, bovine serum albumin (BSA; A7906), ethyl acetate (34858), and butyl 4-hydroxybenzoate (54680) were obtained from Sigma-Aldrich. Wright–Giemsa stain was purchased from Volu-Sol, and Permount mounting medium was obtained from Fisher Scientific. Thrombin was purchased from MP Biomedicals (154163), and prostaglandin E1 was obtained from Tokyo Chemical Industry. An ultrasonic disruptor (Misonix SL-2000) equipped with a microtip probe was used for nanoparticle preparation. Dynamic light scattering (DLS) measurements were performed using a Brookhaven Instruments 90Plus particle size analyzer. BALB/c mice were obtained from Taconic Biosciences (Rensselaer, NY). C57/BL (B6­(Cg)-Tyr < c-2J>/J) mice were from The Jackson Laboratory (Bar Harbor, ME). EMT6 murine mammary carcinoma cells were obtained from the American Type Culture Collection (ATCC). The KPC cell line was kindly provided by Dr. Mario Shields, Pathology Department, Stony Brook University (Stony Brook, NY) which was generated from the KPC (KrasG12D/+; Trp53R172H/+; P48-Cre) mouse model (Jackson Laboratory #014647).

### Methods

#### Fibrinogen–Paclitaxel Nanoparticle (FDN-PTX) Preparation

FDN-PTX was prepared using a sonication-based formulation method. In a typical synthesis, 26.7 mg fibrinogen was dissolved by adding 125 μL of 0.1 M borate buffer pH 8.6, then 50 μL 3 M NaCl, then 660 μL water without mixing and incubated for 10 min at 37 °C, vortexed, then 12 min at 37 °C. Separately, 2.2 mg of PTX was dissolved in 40 μL PEG 200, heated for 10 min at 80 °C, vortexing at 2 and 6 min the fibrinogen solution was placed on ice water and allowed to cool (about 2 min). The PEG–PTX solution was removed from heat and allowed to cool to room temperature. 40 μL of PEG–PTX was added to the bottom of the fibrinogen solution tube (which is in ice water) with a manual pipet. The solution was sonicated in an ice water bath for 3 min at 4.5 power setting. 125 μL of 0.2 M phosphate buffer pH 7.4 (no NaCl) was added to the solution and vortexed. The solution was sonicated for 3 min at 4.5 power (in an ice water bath). The solution was filtered through a 1 μm filter and measured by DLS. For injection, the appropriate volume was used depending on the weight of the mouse.

#### Fibrinogen–Docetaxel Nanoparticle (FDN-DTX) Preparation

FDN-DTX nanoparticles were prepared using a sonication-based formulation method adapted for in vivo dosing and scaled to mouse body weight. For each mouse, a 2× volume was prepared to facilitate sonication of small volumes. Human fibrinogen was dissolved at a dose of 525 mg/kg (2×; e.g., 21 mg for a 20 g mouse) in a mixture of 33 μL 1 M Tris buffer (pH 8.0), 66 μL deionized water, and 200 μL PBS. The solution was incubated for ∼10 min and vortexed to ensure complete dissolution.

Docetaxel (DTX) was dissolved in PEG-200 at 50 mg/mL by heating to 80 °C for 2 min, repeated three times, until fully solubilized. After approximately 20 min, once the fibrinogen solution was fully dissolved, the appropriate volume of DTX solution (corresponding to a final dose of 90 mg/kg, 2×) was added by pipetting to the bottom of the fibrinogen solution. The mixture was vortexed for 10 s and sonicated using a microtip probe sonicator at power level 3 for 3 min in an ice–water bath, taking care to avoid foaming. Following initial sonication, 400 μL PBS was added, and the solution was briefly vortexed and further sonicated at power level 4.5 for 4 min to ensure homogeneous nanoparticle formation. Nanoparticles were used within approximately 20 min of preparation and administered intravenously to deliver final doses of 525 mg/kg fibrinogen and 90 mg/kg docetaxel. Immediately after preparation, nanoparticles exhibited an average diameter of 433 ± 137 nm (polydispersity index [PDI] 0.22 ± 0.06). At 30 min postpreparation, particle size increased to 575 ± 124 nm with a PDI of 0.29 ± 0.05 (n = 6).

#### EMT6 Triple-Negative Breast Tumor Model and Treatment

A syngeneic triple-negative breast cancer model was established using EMT6 murine mammary carcinoma cells. EMT6 cells (1 × 10^6^ tumor cells mixed 1:1 with Matrigel in 30 μL final volume) were implanted subcutaneously into the hindlimbs of immunocompetent mice and allowed to grow until tumors reached the indicated starting volume prior to treatment. For treatment experiments, mice received FDN-PTX prepared as described above. Nanoparticles were administered intravenously at a fibrinogen dose of 525 mg/kg together with the indicated paclitaxel payload. In experiments employing vascular priming, mice received the vascular disrupting agent DMXAA (18 mg/kg, intraperitoneal) shortly before nanoparticle administration. Tumor growth was monitored by caliper measurements and the tumor volume was calculated using the formula: V = 0.5­(length × width^2^). Animals were followed longitudinally for tumor regression, recurrence and survival. Mice were monitored regularly for tumor growth and overall health. Animals were euthanized when tumor volume exceeded 1,000 mm^3^, which was defined as the end point for tumor overgrowth, or if animals exhibited signs of distress, in accordance with institutional animal care guidelines.

#### KPC Pancreatic Tumor Model and Treatment

A syngeneic pancreatic ductal adenocarcinoma model was established using KPC tumor cells derived from Kras^LSL-G12D/+; Trp53^LSL-R172H/+; Pdx1-Cre mice. Tumor cells (2 × 10^6^) were mixed 1:1 with Matrigel in 100 μL final volume and implanted subcutaneously into the hindlimbs of immunocompetent C57/BL mice and allowed to grow until tumors reached the indicated starting volume prior to treatment. For treatment experiments, mice received FDN-PTX or FDN-DTX. Nanoparticles were administered intravenously at a fibrinogen dose of 525 mg/kg together with the indicated taxane payload. Mice received the vascular disrupting agent DMXAA (18 mg/kg, intraperitoneal) 20 min before nanoparticle administration and 10 min after nanoparticle completion. Tumor growth was monitored by caliper measurements and the tumor volume was calculated using the formula: V = 0.5­(length × width^2^). Animals were followed longitudinally for tumor regression, recurrence and survival. Mice were monitored regularly for tumor growth and overall health. Animals were euthanized when tumor volume exceeded 1,000 mm^3^, which was defined as the end point for tumor overgrowth, or if animals exhibited signs of distress, in accordance with institutional animal care guidelines.

#### Clauss Assay

Samples at the fibrinogen concentration indicated were mixed with 75 NIH U/mL thrombin and the absorbance at 405 nm was measured over time.

#### Histology

Tumors were excised 24 h after treatment with VDA only (18 mg/kg, IP) or VDA (18 mg/kg, IP) plus FDNs (525 mg fibrinogen/kg, 44 mg PTX/kg, IV) and placed in formalin. After 48 h the tumors were washed several times with PBS. Immunohistochemistry was performed by Premier Laboratory, LLC, Longmont, CO histology service and stained using antifibrinogen/fibrin antibodies (reactive with bovine and human) and secondary horseradish peroxidase and 3,3′-diaminobenzidine (DAB). Positive and negative controls were done and VDA and VDA+ FDN tumors were processed on the same slide to avoid any differential staining.

#### Drug Delivery and Pharmacokinetics

Tissue and blood samples, collected at specified times after IV injection of nanoparticles, were processed as published,^53^ with minor modifications. Briefly, frozen samples were mixed with 10 volumes of 4% BSA in deionized water and placed in a ball mill (Retsch MM200) for 10 min at 30 Hz. 100 μL of homogenate was then removed and mixed with 10 mL ethyl acetate spiked with 100 μL of butyl 4-hydroxybenzoate (2 ng/μL) in ethyl acetate as an internal standard.^54^ The solution was shaken vigorously and spun at 3,000*g* for 5 min. The organic layer was removed, transferred to a test tube in a 4 °C water bath, and evaporated under a stream of air to complete dryness. The dried residue was reconstituted in 50 μL of acetonitrile, vortexed vigorously, and 50 μL DI water was added. The entire sample (100 μL) was loaded onto an Eclipse XDB-C18 column (5 μm, 3.0 × 150 mm, Agilent, 993967–302) and separated isocratically at 1 mL/min in 1:1 acetonitrile:water using an Agilent 1260 Infinity HPLC system. Peaks were integrated and normalized by the internal standard. Glass materials instead of plastic were used throughout for HPLC experiments.

#### Encapsulation Efficiency

Fifteen milligrams of fibrinogen was dissolved in 1 mL PBS. Paclitaxel (1.5 mg) was dissolved in 40 μL PEG 200 by heating to 80 °C until fully dissolved. The fibrinogen solution was cooled on ice for 15 min before the paclitaxel solution was added. The mixture was vortexed for 5 s, returned to ice, and sonicated at power setting 4.5 for 3 min. An aliquot (500 μL) of the fibrinogen–paclitaxel suspension (containing 0.75 mg paclitaxel) was removed, and 100 mg ammonium sulfate was added. The suspension was vortexed for 2 min to dissolve the ammonium sulfate and precipitate the fibrinogen, followed by centrifugation at 16,000 × g for 2 min. The supernatant was discarded, and the pellet was resuspended in 1 mL ethanol by vortexing and brief sonication, followed by centrifugation at 16,000 × g for 2 min. The ethanol supernatant was collected, diluted 1:50 with ethanol, and the absorbance at 228 nm was measured. A multipoint paclitaxel calibration curve was generated using known paclitaxel standards in ethanol spanning the expected concentration range (Supplementary Figure S3). Sample concentrations were determined by interpolation from the linear calibration curve, and encapsulation efficiency was calculated as the percentage of paclitaxel recovered from the fibrinogen pellet relative to the initial amount of paclitaxel added to the formulation. Drug loading was calculated as the mass of encapsulated paclitaxel divided by the mass of fibrinogen used in the formulation and is reported as mg paclitaxel per mg fibrinogen (wt %). UV–visible spectroscopy was used for encapsulation efficiency measurements because the purified ethanol extracts contained essentially only paclitaxel and therefore did not require chromatographic separation. In contrast, HPLC was used for tissue pharmacokinetic analyses because biological samples contain endogenous compounds that require chromatographic separation for accurate quantification.

#### Drug Release

A method previously described was used with modifications.^56^ Five μL of fibrinogen-PTX nanoparticle solution (containing 11 μg of PTX) was added to 95 μL of 10 mM phosphate buffer, pH 7.0. Ten units of thrombin were added, and the nanoparticle solution was allowed to clot at 37 °C for 45 min. One mL of 4% BSA in 10 mM phosphate buffer was then gently added to the fully clotted solution, mixed completely with a Pasteur pipet, and placed back at 37 °C to begin the release assay. 200 μL of supernatant were removed at 1, 15, 45, and 90 min after BSA addition, centrifuged 2 min at 16,000*g*, and extracted with 5 mL of ethyl acetate and processed as indicated in the drug delivery and pharmacokinetics section.

#### Platelet Aggregation Microscopy Study

Microscope slides were prepared by spreading a 0.1% poly lysine solution and drying with a heat gun to enhance cell adhesion. Blood was freshly drawn from a mouse and mixed with trisodium citrate anticoagulant (85 mM trisodium citrate, 71 mM citric acid, and 111 mM dextrose) in a ratio of 1 part anticoagulant to 9 parts blood. Platelet rich plasma (PRP) was obtained by centrifuging the blood at 200 g for 10 min and taking the supernatant. For assessment of activated platelets, 8 μL of PRP was applied to the slide and 8 μL of FDNs (26 mg/mL fibrinogen, 2 mg/mL PTX) and 1 μL of ADP (0.1 mg/mL) were immediately added and stirred with a pipet tip. The mixed drop was then spread over the slide and allowed to air-dry. Staining was done by adding 6 drops of Wright-Giemsa stain 1 drop at a time with a 10 s delay between drops. A drop of Permount was added and a coverslip applied. Slides were imaged at 1,000x using oil immersion with a Nikon Optiphot-2 microscope with DS-F11 camera and NIS-Elements D3.2 software. For assessment of activation-inhibited platelets, 1 mL of blood was mixed with anticoagulant (as above) and 1 μL of a 1 mg/mL solution of prostaglandin E1. PRP was prepared as above then 8 μL PRP mixed with 8 μL of FDNs and the slide prepared as described above.

#### Transmission Electron Microscopy

Fibrinogen–drug nanoparticles were applied to poly-l-lysine-treated 400-mesh copper grids (Ted Pella, product no. 01822-F). Excess sample was wicked away, and the grids were washed with deionized water and negatively stained with Nano-W (Nanoprobes, product no. 2018). Images were acquired using an FEI BioTwin G2 transmission electron microscope.

#### Fluorescent Fibrinogen Nanoparticles

Fourteen mg bovine fibrinogen (Thermo Scientific J63276.06) was dissolved in 1 mL of 0.1 M phosphate buffer, pH 7.5. 0.4 mg of the lipophilic BODIPY 493/503 (CAS No . 121207–31–6, TCI Chemicals) was dissolved in 50 μL dimethyl sulfoxide (DMSO) and heated for 2 min at 80 °C. When cooled to room temperature, the BODIPY was added to the fibrinogen solution and sonicated in an ice water bath using a microtip sonicator (Misonix Inc., model XL2000) with the tip in the solution at power setting 4.5 for 4 min. A clear transparent orange solution of nanoparticles resulted with a size of 314 nm and polydispersity of 0.28 as measured by dynamic light scattering using a Brookhaven Instruments Corp. 90Plus machine. Some of the solution was diluted 50-fold and exposed to a 365 nm UV lamp (Spectroline model ENF-260C) and photographed.

#### Institutional Animal Assurance

All animal experiments were performed following the protocols evaluated and approved by the Nanoprobes’ Institutional Animal Care and Use Committee (Ethics Approval Numbers: Protocols #1-rev11_21 and #3).

## Supplementary Material



## Notes

Additional materials and methods; transmission electron microscopy images of fibrinogen–drug nanoparticles; paclitaxel calibration curve; and supplementary experimental data (PDF)

## Abbreviations

%ID/g,: percent injected dose per gram
ADP,: adenosine diphosphate
AUC,: area under the curve
BSA,: bovine serum albumin
DLS,: dynamic light scattering
DMXAA,: 5,6-dimethylxanthenone-4-acetic acid
DTX,: docetaxel
FDN,: fibrinogen–drug nanoparticle
FDN-DTX,: fibrinogen–docetaxel nanoparticle
FDN-PTX,: fibrinogen–paclitaxel nanoparticle
GPIIb/IIIa,: glycoprotein IIb/IIIa integrin
IC_50_,: half maximal inhibitory concentration
IP,: intraperitoneal
IV,: intravenous
KPC,: Kras^LSL-G12D/+; Trp53^LSL-R172H/+; Pdx1-Cre pancreatic cancer model
PBS,: phosphate-buffered saline
PDAC,: pancreatic ductal adenocarcinoma
PDI,: polydispersity index
PEG,: polyethylene glycol
PRP,: platelet-rich plasma
PTX,: paclitaxel
SEM,: standard error of the mean
TNBC,: triple-negative breast cancer
V,: tumor volume
VDA,: vascular disrupting agent

## References

[1] Peer D., Karp J. M., Hong S., Farokhzad O. C., Margalit R., Langer R. (2007). Nanocarriers
as an Emerging Platform
for Cancer Therapy. Nat. Nanotechnol..

[2] Minchinton A. I., Tannock I. F. (2006). Drug Penetration in Solid Tumours. Nat. Rev. Cancer.

[3] Cheng Y.-H., He C., Riviere J. E., Monteiro-Riviere N. A., Lin Z. (2020). Meta-Analysis of Nanoparticle
Delivery to Tumors Using a Physiologically Based Pharmacokinetic Modeling
and Simulation Approach. ACS Nano.

[4] Wilhelm S., Tavares A. J., Dai Q., Ohta S., Audet J., Dvorak H. F., Chan W. C. W. (2016). Analysis of Nanoparticle Delivery
to Tumours. Nat. Rev. Mater..

[5] Kopp A., Hofsess S., Cardillo T. M., Govindan S. V., Donnell J., Thurber G. M. (2023). Antibody-Drug Conjugate
Sacituzumab Govitecan Drives
Efficient Tissue Penetration and Rapid Intracellular Drug Release. Mol. Cancer Ther..

[6] Bonaventura P., Shekarian T., Alcazer V., Valladeau-Guilemond J., Valsesia-Wittmann S., Amigorena S., Caux C., Depil S. (2019). Cold Tumors:
A Therapeutic Challenge for Immunotherapy. Front.
Immunol..

[7] Zabransky D. J., Yarchoan M., Jaffee E. M. (2023). Strategies
for Heating Up Cold Tumors
to Boost Immunotherapies. Ann. Rev. Cancer Biol..

[8] Loganzo F., Sung M., Gerber H.-P. (2016). Mechanisms of Resistance to Antibody-Drug
Conjugates. Mol. Cancer Ther..

[9] von
Maltzahn G., Park J.-H., Lin K. Y., Singh N., Schwoppe C., Mesters R., Berdel W. E., Ruoslahti E., Sailor M. J., Bhatia S. N. (2011). Nanoparticles That Communicate in
Vivo to Amplify Tumour Targeting. Nature Mater.

[10] Song W., Tang Z., Zhang D., Li M., Gu J., Chen X. (2016). A Cooperative Polymeric Platform
for Tumor-Targeted Drug Delivery. Chem. Sci..

[11] Doolittle R. F. (2009). Step-by-Step
Evolution of Vertebrate Blood Coagulation. Cold
Spring Harb Symp. Quant Biol..

[12] Shin E.-K., Park H., Noh J.-Y., Lim K.-M., Chung J.-H. (2017). Platelet
Shape Changes and Cytoskeleton Dynamics as Novel Therapeutic Targets
for Anti-Thrombotic Drugs. Biomol. Ther..

[13] Mozafarpour R., Koocheki A., Sani M. A., McClements D. J., Mehr H. M. (2022). Ultrasound-Modified Protein-Based Colloidal Particles:
Interfacial Activity, Gelation Properties, and Encapsulation Efficiency. Adv. Colloid Interface Sci..

[14] Narayanan S. (1999). Multifunctional
Roles of Thrombin. Ann. Clin. Lab Sci..

[15] Stang L. J., Mitchell L. G. (2013). Fibrinogen. Methods Mol. Biol..

[16] Siemann D. W., Horsman M. R. (2009). Vascular Targeted Therapies in Oncology. Cell Tissue Res..

[17] Tozer G. M., Kanthou C., Lewis G., Prise V. E., Vojnovic B., Hill S. A. (2008). Tumour Vascular
Disrupting Agents: Combating Treatment
Resistance. Br J. Radiol..

[18] Tozer G. M., Kanthou C., Baguley B. C. (2005). Disrupting
Tumour Blood Vessels. Nat. Rev. Cancer.

[19] Lovich M.
A., Creel C., Hong K., Hwang C., Edelman E. R. (2001). Carrier
Proteins Determine Local Pharmacokinetics and Arterial Distribution
of Paclitaxel. J. Pharm. Sci..

[20] Torres M.
P., Rachagani S., Souchek J. J., Mallya K., Johansson S. L., Batra S. K. (2013). Novel Pancreatic Cancer Cell Lines Derived from Genetically
Engineered Mouse Models of Spontaneous Pancreatic Adenocarcinoma:
Applications in Diagnosis and Therapy. PLoS
One.

[21] Ariston
Gabriel A. N., Jiao Q., Yvette U., Yang X., Al-Ameri S. A., Du L., Wang Y., Wang C. (2020). Differences
between KC and KPC Pancreatic Ductal Adenocarcinoma Mice Models, in
Terms of Their Modeling Biology and Their Clinical Relevance. Pancreatology.

[22] Pham T. N. D., Shields M. A., Spaulding C., Principe D. R., Li B., Underwood P. W., Trevino J. G., Bentrem D. J., Munshi H. G. (2021). Preclinical
Models of Pancreatic Ductal Adenocarcinoma and Their Utility in Immunotherapy
Studies. Cancers.

[23] Buey R. M., Barasoain I., Jackson E., Meyer A., Giannakakou P., Paterson I., Mooberry S., Andreu J. M., Díaz J. F. (2005). Microtubule
Interactions with Chemically Diverse Stabilizing Agents: Thermodynamics
of Binding to the Paclitaxel Site Predicts Cytotoxicity. Chem. Biol..

[24] Madani F., Esnaashari S. S., Mujokoro B., Dorkoosh F., Khosravani M., Adabi M. (2018). Investigation of Effective Parameters on Size of Paclitaxel Loaded
PLGA Nanoparticles. Adv. Pharm. Bull..

[25] Grünberg J., Novak-Hofer I., Honer M., Zimmermann K., Knogler K., Bläuenstein P., Ametamey S., Maecke H. R., Schubiger P. A. (2005). In Vivo Evaluation of 177Lu- and 67/64Cu-Labeled Recombinant
Fragments of Antibody chCE7 for Radioimmunotherapy and PET Imaging
of L1-CAM-Positive Tumors. Clin. Cancer Res..

[26] Acute Toxicity of Polyethylene Glycol 200 in Laboratory Animals; https://apps.dtic.mil/sti/citations/ADA106519. (accessed 2026–06–28).

[27] Parise L. V., Phillips D. R. (1985). Reconstitution of the Purified Platelet Fibrinogen
Receptor. Fibrinogen Binding Properties of the Glycoprotein IIb-IIIa
Complex. J. Biol. Chem..

[28] Ducheyne, P. ; Healy, K. ; Hutmacher, D. E. ; Grainger, D. W. ; Kirkpatrick, C. J. Comprehensive Biomaterials II; Elsevier Science: Saint Louis, 2017.

[29] Floyd C. N., Ferro A. (2012). The Platelet Fibrinogen Receptor:
From Megakaryocyte to the Mortuary. JRSM Cardiovasc.
Dis..

[30] Estevez B., Du X. (2017). New Concepts and Mechanisms
of Platelet Activation Signaling. Physiology.

[31] Leuschner F., Nahrendorf M. (2011). Molecular Imaging of Coronary Atherosclerosis and Myocardial
Infarction. Circ. Res..

[32] Festjens N., Vanden Berghe T., Vandenabeele P. (2006). Necrosis, a Well-Orchestrated Form
of Cell Demise: Signalling Cascades, Important Mediators and Concomitant
Immune Response. Biochim. Biophys. Acta.

[100] Ernsting M. J., Murakami M., Undzys E., Aman A., Press B., Li S.-D. (2012). A Docetaxel-Carboxymethylcellulose
Nanoparticle Outperforms the Approved Taxane Nanoformulation, Abraxane,
in Mouse Tumor Models with Significant Control of Metastases. J Control Release.

[54] Yang T., Cui F.-D., Choi M.-K., Cho J.-W., Chung S.-J., Shim C.-K., Kim D.-D. (2007). Enhanced
Solubility and Stability
of PEGylated Liposomal Paclitaxel: In Vitro and in Vivo Evaluation. Int. J. Pharm..

[33] Goldenberg D. M., Stein R., Sharkey R. M. (2018). The Emergence
of Trophoblast Cell-Surface
Antigen 2 (TROP-2) as a Novel Cancer Target. Oncotarget.

[34] Liebmann J. E., Cook J. A., Lipschultz C., Teague D., Fisher J., Mitchell J. B. (1993). Cytotoxic Studies
of Paclitaxel (Taxol) in Human Tumour
Cell Lines. Br. J. Cancer.

[35] Seshadri M., Spernyak J. A., Maiery P. G., Cheney R. T., Mazurchuk R., Bellnier D. A. (2007). Visualizing the
Acute Effects of Vascular-Targeted
Therapy in Vivo Using Intravital Microscopy and Magnetic Resonance
Imaging: Correlation with Endothelial Apoptosis, Cytokine Induction,
and Treatment Outcome. Neoplasia.

[36] Emran T. B., Shahriar A., Mahmud A. R., Rahman T., Abir M. H., Siddiquee M. F.-R., Ahmed H., Rahman N., Nainu F., Wahyudin E. (2022). Multidrug Resistance in Cancer: Understanding
Molecular Mechanisms, Immunoprevention and Therapeutic Approaches. Front. Oncol..

[37] Daei
Farshchi Adli A., Jahanban-Esfahlan R., Seidi K., Samandari-Rad S., Zarghami N. (2018). An Overview on Vadimezan (DMXAA): The Vascular Disrupting
Agent. Chem. Biol. Drug Des..

[38] Cai S. (2007). Small Molecule
Vascular Disrupting Agents: Potential New Drugs for Cancer Treatment. PRA.

[39] Liang W., Ni Y., Chen F. (2016). Tumor Resistance to
Vascular Disrupting Agents: Mechanisms,
Imaging, and Solutions. Oncotarget.

[40] Downey C. M., Aghaei M., Schwendener R. A., Jirik F. R. (2014). DMXAA Causes Tumor
Site-Specific Vascular Disruption in Murine Non-Small Cell Lung Cancer,
and like the Endogenous Non-Canonical Cyclic Dinucleotide STING Agonist,
2’3′-cGAMP, Induces M2Macrophage Repolarization. PLoS One.

[41] Le
Naour J., Zitvogel L., Galluzzi L., Vacchelli E., Kroemer G. (2020). Trial Watch: STING Agonists in Cancer Therapy. Oncoimmunology.

[42] Hallahan D., Geng L., Qu S., Scarfone C., Giorgio T., Donnelly E., Gao X., Clanton J. (2003). Integrin-Mediated
Targeting
of Drug Delivery to Irradiated Tumor Blood Vessels. Cancer Cell.

[43] Fajardo L. F., Prionas S. D. (1994). Endothelial Cells and Hyperthermia. Int. J. Hyperthermia.

[44] Seidl M., Steinbach P., Hofstädter F. (1994). Shock Wave Induced Endothelial Damage—In
Situ Analysis by Confocal Laser Scanning Microscopy. Ultrasound Med. Biol..

[45] Amouzegar A., Chelvanambi M., Filderman J. N., Storkus W. J., Luke J. J. (2021). STING Agonists
as Cancer Therapeutics. Cancers.

[46] Hicks K. C., Chariou P. L., Ozawa Y., Minnar C. M., Knudson K. M., Meyer T. J., Bian J., Cam M., Schlom J., Gameiro S. R. (2021). Tumour-Targeted Interleukin-12 and
Entinostat Combination
Therapy Improves Cancer Survival by Reprogramming the Tumour Immune
Cell Landscape. Nat. Commun..

[47] Ngwa W., Irabor O. C., Schoenfeld J. D., Hesser J., Demaria S., Formenti S. C. (2018). Using Immunotherapy
to Boost the Abscopal Effect. Nat. Rev. Cancer.

[48] Meng F., Wang J., He Y., Cresswell G. M., Lanman N. A., Lyle L. T., Ratliff T. L., Yeo Y. (2022). A Single Local
Delivery of Paclitaxel and Nucleic Acids via an Immunoactive Polymer
Eliminates Tumors and Induces Antitumor Immunity. Proc. Natl. Acad. Sci. U. S. A..

[49] Abdelrahim M., Konduri S., Basha R., Philip P. A., Baker C. H. (2010). Angiogenesis:
An Update and Potential Drug Approaches (Review). Int. J. Oncol.

[50] Vilar R., Fish R. J., Casini A., Neerman-Arbez M. (2020). Fibrin­(Ogen)
in Human Disease: Both Friend and Foe. Haematologica.

[51] Glas G. J., Levi M., Schultz M. J. (2016). Coagulopathy and Its Management in
Patients with Severe Burns. J. Thromb. Haemost..

[52] Silva A. V., Norinder U., Liiv E., Platzack B., Öberg M., Törnqvist E. (2021). Associations
between Clinical Signs and Pathological
Findings in Toxicity Testing. ALTEX.

[53] Kim S. C., Yu J., Lee J. W., Park E.-S., Chi S.-C. (2005). Sensitive HPLC Method
for Quantitation of Paclitaxel (Genexol in Biological Samples with
Application to Preclinical Pharmacokinetics and Biodistribution. J. Pharm. Biomed. Anal..

[56] Abouelmagd S. A., Sun B., Chang A. C., Ku Y. J., Yeo Y. (2015). Release Kinetics Study
of Poorly Water-Soluble Drugs from Nanoparticles: Are We Doing It
Right?. Mol. Pharmaceutics.

