# Supplementary material for: Fibrinogen–Drug Nanoparticles Achieve Durable Responses in Pancreatic and Breast Cancer Murine Models
Source: ACS Pharmacol Transl Sci. 2026 Aug 21;9(9):2628–38. doi: 10.1021/acsptsci.6c00337 (PMC13632883; doi:10.1021/acsptsci.6c00337)
Supplement: Supplementary file 1 [file pt6c00337_si_001.pdf]

Supporting Information for

Fibrinogen-Drug Nanoparticles Achieve Durable Responses in Pancreatic and Breast Cancer Murine Models

Rose Razavi[1 2 3], Michael O'Connor[1], James F. Hainfeld[1*]

Affiliations:

[1]Nanoprobes, Inc.; Yaphank, NY 11980, USA.

[2]Penn Institute for RNA Innovation, Perelman School of Medicine, University of Pennsylvania; Philadelphia, PA 19104, USA.

[3]Department of Systems Pharmacology and Translational Therapeutics, Perelman School of Medicine; University of Pennsylvania, Philadelphia, PA 19104, USA.

*Email: hainfeld@nanoprobes.com

Table of Contents

Figure S1. Paclitaxel-loaded fibrinogen nanoparticles …….. S-2
Figure S2. Transmission electron microscopy ……………… S-3
Figure S3. Paclitaxel calibration curve …………………. S-3

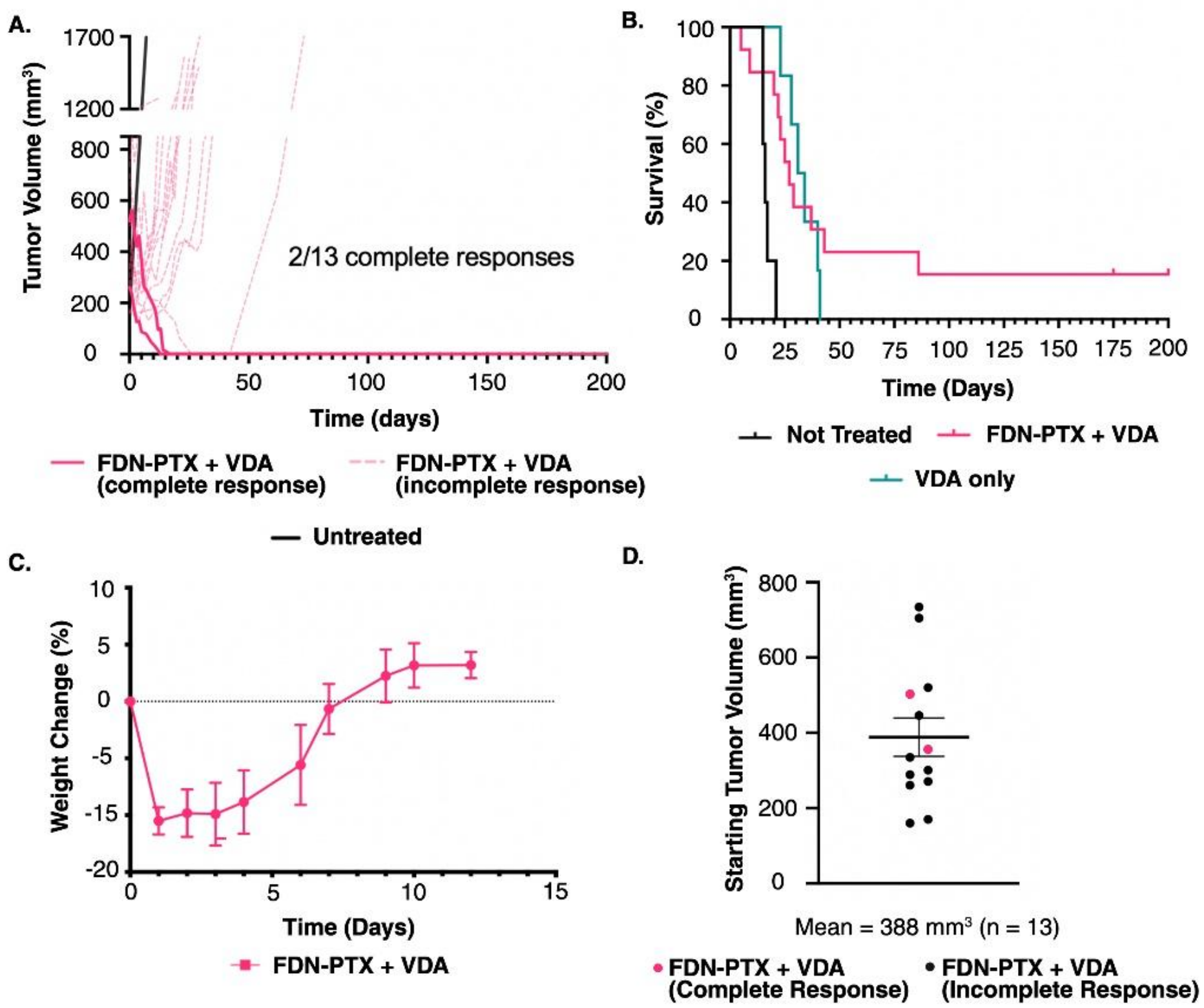

Fig. S1 Paclitaxel-loaded fibrinogen nanoparticles produce heterogeneous responses in KPC pancreatic tumors. (A) Individual tumor growth trajectories following treatment with fibrinogen–paclitaxel nanoparticles (FDN-PTX) combined with a vascular disrupting agent (VDA) in KPC tumor–bearing mice (n = 13). Two of thirteen treated animals exhibited complete tumor regression (solid pink lines), whereas the remaining animals showed partial or transient responses followed by tumor progression (dashed pink lines). Representative untreated tumor growth is shown for comparison. **(B)** Kaplan–Meier survival analysis corresponding to panel A. VDA + FDN-PTX (n=13) improved survival relative to not treated controls (n = 5), and VDA only controls (n=6) but did not produce consistent durable tumor eradication. **(C)** Body weight changes following treatment with FDN-PTX + VDA (n = 13). Combination therapy produced a transient decrease in body weight during the first week followed by recovery to baseline. **(D)** Distribution of starting tumor volumes in the FDN-PTX + VDA cohort (n = 13). Tumors ranged broadly in size at treatment initiation (mean = 388 mm³). Pink points denote mice that achieved complete tumor regression. Created in BioRender. Razavi, R. (2026) https://BioRender.com/94cuaim

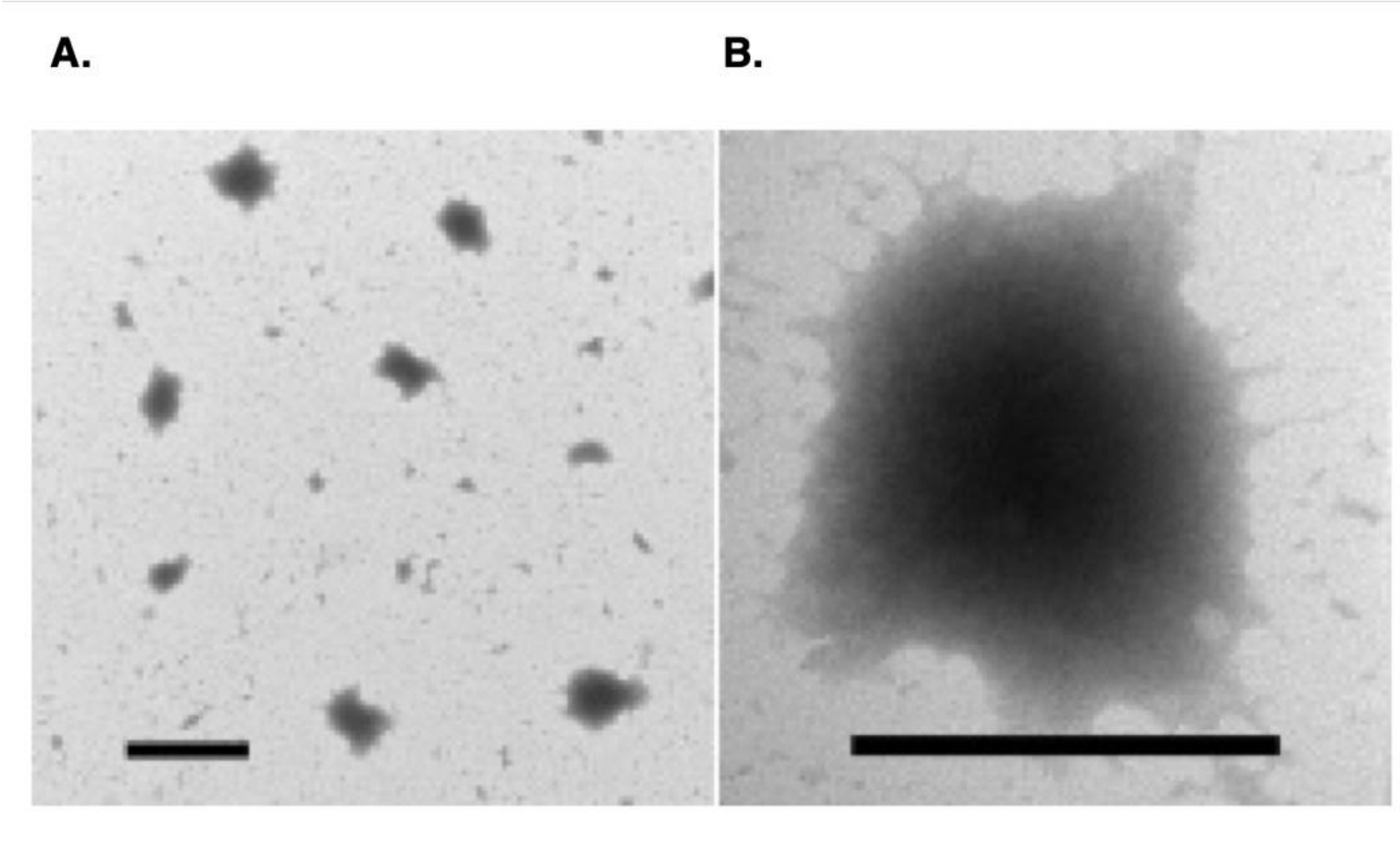

Figure S2. Transmission electron microscopy of fibrinogen–drug nanoparticles (FDNs). Representative TEM images of negatively stained FDNs at **(A)** lower and **(B)** higher magnification. Scale bars: **(A)** 500 nm and **(B)** 300 nm. Dynamic light scattering measured a mean particle size of 322 nm with a polydispersity index (PDI) of 0.16. https://BioRender.com/8cw7520

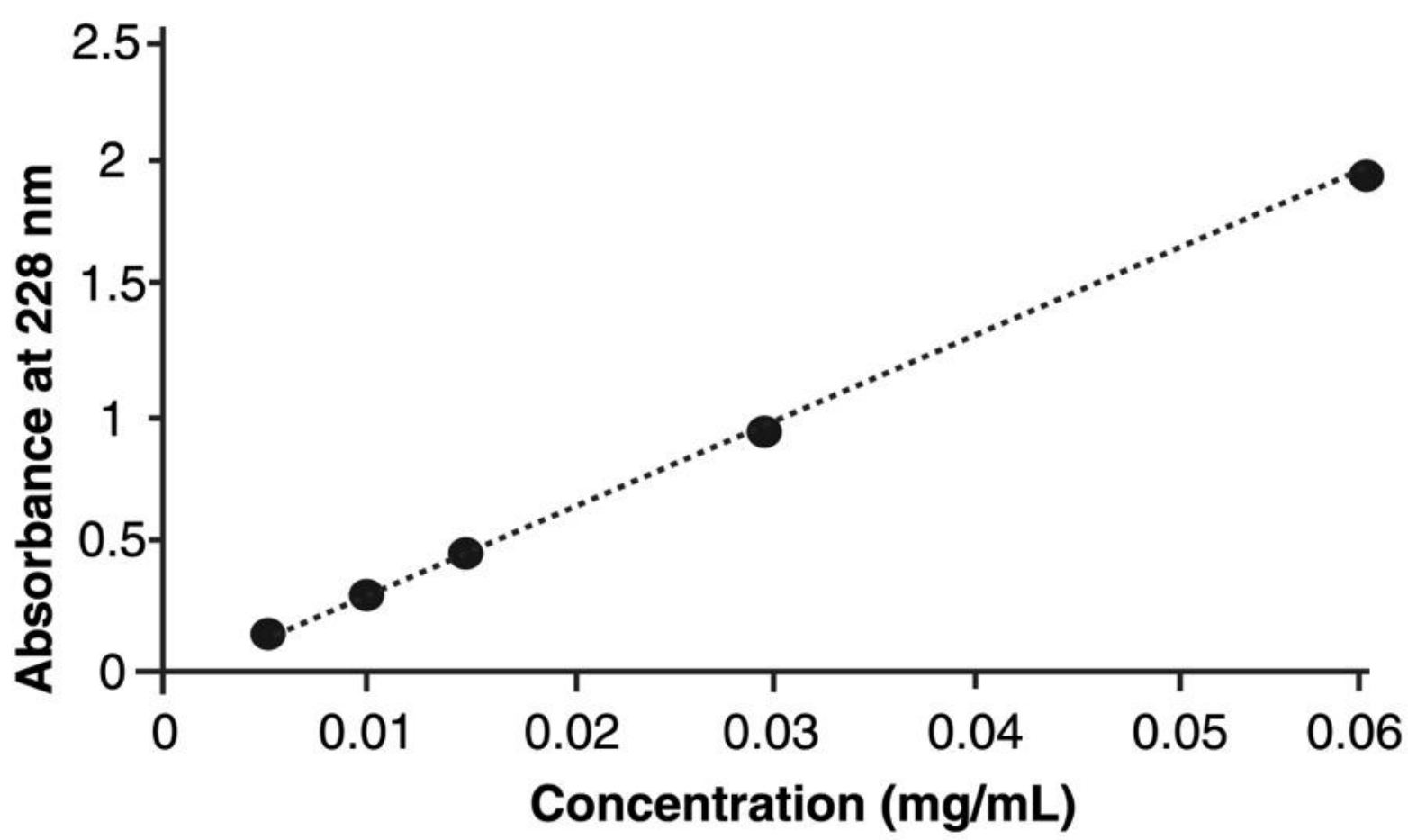

Figure S3. Paclitaxel calibration curve for UV spectrophotometric quantification. Calibration standards (0.005–0.060 mg/mL) were analyzed at 228 nm, yielding a linear relationship between absorbance and concentration ($y = 32.22x + 0.0267$, $R^2 = 0.9993$). This calibration curve was used to quantify paclitaxel for encapsulation efficiency and drug loading analyses. https://BioRender.com/s8scou9
